# Predicting Gilthead Sea Bream (*Sparus aurata*) Freshness by a Novel Combined Technique of 3D Imaging and SW-NIR Spectral Analysis

**DOI:** 10.3390/s16101735

**Published:** 2016-10-19

**Authors:** Eugenio Ivorra, Samuel Verdu, Antonio J. Sánchez, Raúl Grau, José M. Barat

**Affiliations:** 1 Departamento de Ingeniería de Sistemas y Automática, Universidad Politècnica de València, Valencia 46022, Spain; asanchez@isa.upv.es; 2 Departamento de Tecnología de Alimentos, Universidad Politècnica de València, Valencia 46022, Spain; saveram@upvnet.upv.es (S.V.); rgraume@tal.upv.es (R.G.); jmbarat@tal.upv.es (J.M.B.)

**Keywords:** hyperspectral imaging, 3D segmentation, 3D structured light, SW-NIR, fish freshness

## Abstract

A technique that combines the spatial resolution of a 3D structured-light (SL) imaging system with the spectral analysis of a hyperspectral short-wave near infrared system was developed for freshness predictions of gilthead sea bream on the first storage days (Days 0–6). This novel approach allows the hyperspectral analysis of very specific fish areas, which provides more information for freshness estimations. The SL system obtains a 3D reconstruction of fish, and an automatic method locates gilthead’s pupils and irises. Once these regions are positioned, the hyperspectral camera acquires spectral information and a multivariate statistical study is done. The best region is the pupil with an R^2^ of 0.92 and an RMSE of 0.651 for predictions. We conclude that the combination of 3D technology with the hyperspectral analysis offers plenty of potential and is a very promising technique to non destructively predict gilthead freshness.

## 1. Introduction

Annual fish consumption in Europe in 2013 was 23.2 kg [1], which gives an idea of just how big the fishing industry is. As in all food industries, product safety and quality are top priorities in the fishing industry. Fish freshness is extremely important [2] because fishery products are highly perishable and spoil quickly in only a few days if not submitted to a food preservation process. Hence their shelf life is short. The shelf life of fish is defined as the complete period during which fish is considered fit for human consumption [3]. Therefore, the shelf life of fish is a critical quality aspect that must be assured. The European Commission established a sensory assessment method to evaluate such quality (Council Directive 95/149/EEC, March 1995), known as the Quality Index Method (QIM). In case of doubt, techniques like Total Volatile Basic Nitrogen (TVB-N) and microbiological tests could be used. However, these methods are subjective if a sensory method is followed, and are destructive in TVB-N and microbiological tests. Moreover, they are all expensive and time-consuming, and none provide freshness estimations on the first storage days. Changes in physicochemical and microbiological properties for the first three days after sacrifice are minimum, which makes it difficult to estimate when fish were sacrificed [4,5]. Hence a method is needed that measures freshness on the first few days to monitor the fish that arrive at markets from capture areas or fish farms, and to also evaluate fish one by one in an on-line procedure.

Gilthead sea bream (*Sparus aurata*) is one of the main farmed fish species in the Mediterranean region, whose production was estimated to be 138,694 tons in 2012 [6]. In fact more than 700 papers have been published since 2012 about this fish species. Currently a great deal of interest is being shown in developing a method for gilthead freshness estimations. This would explain the large number of related works and different techniques employed; including chemical, physical and sensory methods [7]; potentiometric sensor [8], torrymeters [9], optoelectronic nose [10], impedance spectroscopy [4], machine vision [11] and hyperspectral imaging [12]. Of all these methods, the most suitable are those based on machine vision and hyperspectral systems because they are fast, cheap, non destructive, require minimum sample manipulation and can evaluate more quality features, such as visual appearance or fish size. Specifically, hyperspectral imaging has proven an effective tool for quality analyses, for controlling fish [13] and for evaluating freshness on the first storage days. Some examples are the recent studies of fish freshness assessments by hyperspectral imaging published by Cheng et al. [14,15] in grass carp fillets, or of Ivorra et al. [16,17] in smoked salmon.

It is noteworthy that fish have different kinds of tissues that present various biodegradation rates, which have to be taken into account when assessing freshness [16,18]. In fact Dowlati et al [11] and Menessati et al [12] have identified specific fish regions that correlate more with storage time. Some of these regions are fish eyes and the posterior opercular spine. Thus a spectral study was conducted in these regions. The eye region was segmented into pupils and irises, which were separately analyzed. Automatic landmarking and spatial segmentation can be performed by diverse methods; e.g., using the same hyperspectral system [16] or an independent visible 2D camera [11]. In this work, an active 3D system based on structured light [19,20,21] was employed to take into account 3D information, and to achieve accurate, automatic and robust spatial segmentation for tracking fish eyes.

In the last decade, three-dimensional information sensors with hyperspectral sensors to maximize both technologies have merged. These combinations have been used to primarily study the distribution of vegetation biomass [22], for tree classifications [23] from aerial reconnaissance devices, or in the geology field for studying mineral outcrops [24,25]. Currently, we are unaware of any study conducted in the food field that has combined 3D imaging technologies with hyperspectral information.

Our study objective was to achieve a novel technique that combines a robust 3D spatial segmentation of fish with an accurate SW-NIR spectroscopy hyperspectral imaging analysis of the selected regions to predict the freshness of gilthead sea bream on the first storage days.It is important to clarify that throughout this paper the term “freshness” is simplified as days elapsed since a sample was harvested, by assuming that samples were stored at 4 °C.

## 2. Materials and Methods

### 2.1. Sample Preparation

The study was carried out on 48 *Sparus aurata* samples (12 samples from four different fish fattening cages each) acquired from a fish farm (Culmarex, S.A.U., Murcia, Spain). Samples, which were submitted to the same feeding and treatment conditions, were simultaneously sacrificed by immersing in ice and obtaining 24-h post-mortems. Sample weights were between 400 g and 500 g.

Images were captured on Days 0, 1, 3 and 6. On each sampling day, two samples were used for the destructive analyses. The objective of these analyses was to monitor samples evolution to guarantee an experiment without anomalies. Fish were scanned on both sides with the hyperspectral camera and the 3D system at room temperature (21 °C). Fish samples remained at room temperature when acquiring the spectrum of samples. After acquisition, samples were packed and stored at 4 °C. A single storage temperature was used to simplify the experimental design. However as some authors have suggested [26,27], the developed technique will be tested with samples with different storage conditions as a future work.

#### 2.1.1. Destructive Analyses

During the freshness study, quality was evaluated by measuring pH, Total Volatile Basic Nitrogen (TVB-N) and the refractive index (RI) of vitreous humor. The pH of samples was measured with a pH-meter MM40 (Crison Instruments S. A, Alella, Barcelona, Spain). Measuring was done by dipping the pH electrode into a mixture of homogenized sample and distilled water (10 g of homogenized fish meat and 90 mL of distilled water) [28]. In order to measure TVB-N, 10 g of sample were washed in the distillation tube and 1 g of magnesium oxide was added. Samples were distilled into 10 mL of HCl solution with the included indicator. The resultant solutions were titrated with a NaOH solution, 0.1 N. The results were expressed as mg of TVB-N/100 g [29]. The RI of vitreous humor was evaluated by extracting the vitreous humor from eyes with a syringe. Its RI was analyzed by an ABBE refractometer [30].

In order to evaluate the initial and final microbiological statuses, mesophilic counts were taken according to the method featured in ISO Standard 4833:2003 (ISO, 2003). Enterobacteriaceae were enumerated according to the method described by Pascual and Calderón (2000).

#### 2.1.2. Imaging System

**Figure 1:**
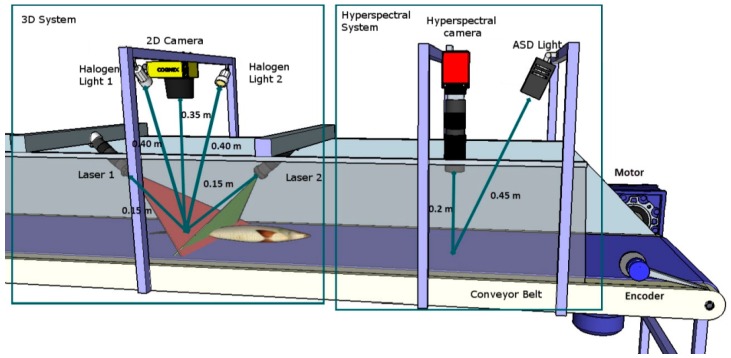
The 3D and hyperspectral acquisition system for gilthead freshness estimations.

The imaging system comprised two subsystems (Figure 1): a structured-light (SL) 3D system, which provides very accurate spatial information; and a hyperspectral acquisition system, which supplies high-resolution spectral information. By employing both subsystems, it was possible to recognize and identify the areas to be studied and to analyze these areas. Subsystems were mounted on a Nikatrans NT130 conveyor belt (Nikai Systems S.L., Guadalajara, Spain), driven by a Nord 71s/4 AC motor (NordDrivesystems SL, Bargteheide, Germany), and were controlled by a Telemecanique Altivar 31 inverter (Schneider Electric, Rueil-Malmaison, France). Speed was measured with a Wachendorff WDG LMS encoder (Wachendorff Automation, Geisenheim, Germany).

(1) 3D SL Acquisition System

The structured-light method is based on projecting a pattern of light onto a sample and calculating the 3D dimensions from the deformation of the pattern with a camera. The patterns used herein were two parallel lines projected by two red line lasers (Lasiris SNF 410, Coherent Inc., Santa Clara, CA, USA), with one placed in front of the other to reduce hidden areas. The camera (AD-080CL, JAI Company, Yokohama, Kanagawa, Japan), which worked at 15 fps, was placed 0.035 m above the conveyor belt, and was also positioned so that the line lasers projected onto two different and single rows in the image. Both the laser and camera were fixed, and 3D geometry was achieved by moving the sample along the conveyor belt at a constant speed of 15 × 10^−4^ m/s. The conveyor belt could be speeded up to industrials levels by using a high-speed camera or by reducing the 3D resolution.

The equipment was calibrated by taking 10 regularly distributed points in 3D on the laser projection plane [31]. A homography transformation was calculated by using these points with known 3D coordinates and their corresponding points on the image [32].

The laser points projected onto the image were extracted by following the steps in the method as so [20,33]: first, the image was segmented using Otsu’s global threshold [34], then it was filtered by removing the connected components with an area lower than 100 pixels. Finally, row coordinates were calculated by the weighted mean. This weighted mean value was calculated for each column using the intensity value to accomplish subpixel precision. The 3D coordinates were then calculated by using the homography and by applying a rotation matrix to make the *Z*-axis normal to the conveyor belt surface. This process was repeated for both lasers and two incomplete 3D meshes were obtained. In order to accomplish a complete reconstruction, these meshes were merged by averaging the 3D overlapped points.

Image processing methods were calculated by our own code, which was developed with Matlab R2013a (The Mathworks, Natick, MA, USA).

(2) Hyperspectral Acquisition and Preprocessing

Image acquisition was performed by using a Photonfocus MV1-D1312 40 GB 12 CMOS camera (Photonfocus AG, Lachen, Switzerland) and a SpecimImSpector V10 1/2″ filter (Specim Spectral Imaging, LTD., Oulu, Finland), which works as a linear hyperspectral camera. Stable illumination was achieved across the full spectral working range with an ASD illuminator reflectance lamp (ASD Inc., Boulder, CO, USA).

The position of both the illuminant and camera to the sample was left unchanged to maintain constant lighting and to not alter the relationship with the 3D system. The distance between the illuminant and the sample was 0.525 m, while that between the camera and the sample was 0.225 m. The obtained image (scanned line) was composed of a 256-level (8-bit) gray scale. The diffuse reflectance spectrum was acquired within the 400–1000 nm range using 53 different wavelengths at intervals of 11.2 nm. The spatial resolution was 1312 pixels, which gave a resolution of 1 pixel/mm in the working distance.

The camera was operated by our own software, which was developed based on SDK Photonfocus-GigE_Tools using the C++ programming language.

Reflectance calibration was performed to normalize the non inear light source reflectance. This was done by applying Equation (1), where $r_{w}$ is the reflectance value of white pattern reflectance acquired under the same conditions, $r_{D}$ denotes the dark current, measured by covering the camera’s objective, and $r_{s}$ is sample reflectance:

(1) $$R\left(\lambda \right)=\frac{\left(r_{s}-r_{D}\right)}{\left(r_{w}-r_{D}\;\right)}$$

The other operations ran on the spectra for further statistical processing purposes included mean-centering, unit variance normalization, and orthogonal signal correction (OSC) which removes any orthogonal variance to the Y-block (measured variables) and ensures that signal correction removes as little information from Y as possible [35].

Image reflectance calibration and preprocessing were performed by our own code, developed in Matlab R2013a (The Mathworks, Natick, MA, USA).

(3) Synchronization between the Two Subsystems

Synchronization between the two subsystems and the conveyor belt was achieved using a computer to calculate when and where information was to be acquired. This calculation consisted in transforming the located 3D points of interest using the 3D system into a world system coordinate and then estimating when these points would be in a position to be acquired. Finally these 3D points, expressed in the world coordinate system, were transformed into the image pixels of the hyperspectral system so that the computer could trigger the system’s image acquisition and keep only the interested pixels for the spectral analysis. A specific example is provided in mathematical details below.

**Figure 2:**
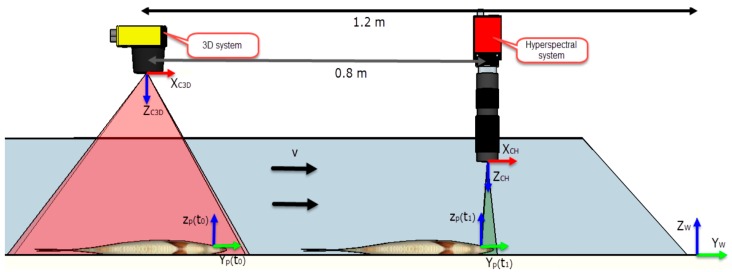
The 3D and hyperspectral systems schematic.

The starting point was to place the coordinate systems as shown in the schematics of Figure 2. Equations (2) and (3) are homogeneous transformation matrices that define the location of the world coordinate system (*W*) in relation to the coordinate systems of cameras *C3D* and *CH*. These camera coordinate systems are at their respective optical centers with the *Z*-axis on their optical axis camera.

(2) $${}^{w}T_{C3D}=\left[\begin{array}{cc} R_{3\times 3} & {}^{w}t_{C3D,3\times 1}\\ 0_{1\times 3} & 1 \end{array}\right]=\left[\begin{array}{cccc} 0 & 1 & 0 & -0.3\\ 1 & 0 & 0 & -1.2\\ 0 & 0 & 1 & 0.35\\ 0 & 0 & 0 & 1 \end{array}\right]$$

(3) $${}^{w}T_{CH}=\left[\begin{array}{cc} R_{3\times 3} & {}^{w}t_{CH,3\times 1}\\ 0_{1\times 3} & 1 \end{array}\right]=\left[\begin{array}{cccc} 0 & 1 & 0 & -0.3\\ 1 & 0 & 0 & -0.4\\ 0 & 0 & 1 & 0.2\\ 0 & 0 & 0 & 1 \end{array}\right]$$

where **R** is the 3 × 3 rotation matrix that defines the orientation of the cameras regarding *W*; *^w^t_CH_*, *^w^t_C3D_* is the column vector 3 × 1 that defines where the cameras are regarding *W*, and *0* is void row vector 1 × 3.

By knowing *^w^***T***_C3D_*, and *^w^***T***_CH_*, the inverse matrices were calculated so that a point expressed in the coordinate system of the 3D system (*^C3D^p*) could be transformed into the world system, and later to the hyperspectral system using Equation (4).

(4) $${}^{w}p{=}^{w}T_{C3D}\cdot^{C3D}p;{}^{CH}p{=}^{CH}T_{w}\cdot^{w}p{=}^{CH}T_{w}\cdot^{w}T_{C3D}\cdot^{C3D}p$$

The instant of time where the desired 3D point falls within the range of the hyperspectral system can be determined by Equation (5). The position of a point at a specific time can be calculated using the known conveyor belt speed *v* (given by the enconder) by assuming that the direction along the *Y_w-_*axis does not change and that fish do not move on the *X_w-_*axis by Equation (6).

(5) $$t_{1}-t_{0}=\frac{{}^{w}o_{CH}{-}^{w}p'(t_{0})}{v}$$

where *^w^O_CH_* is the projected point of the origin of the hyperspectral coordinate system on the conveyor belt and ${}^{w}p'(t_{0})$ is the desired 3D point whose *Z* coordinate is set at zero.

(6) $${}^{w}p'(t_{1})=v\cdot (t_{1}-t_{0})$$

Finally, projective matrix P, which transforms the desired 3D points expressed in the hyperspectral coordinate system (*^CH^p*) into the pixels points of the hyperspectral camera, was calculated by the calibration method of [36] for calibrating linear cameras, where only a wavelength of the visible range was used (700 nm).

### 2.2. Data Processing

**Figure 3:**
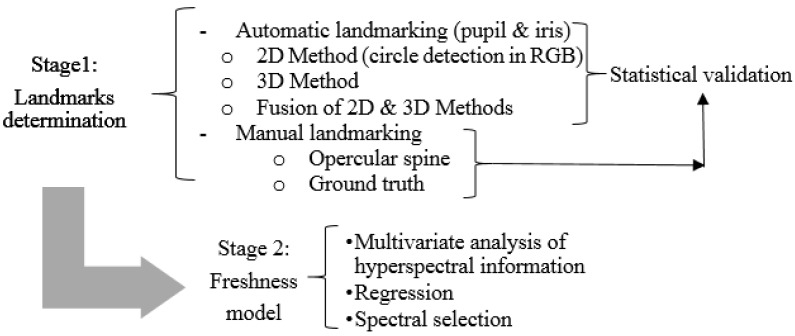
Schematic diagram of the method developed to predict shelf life in *Sparus aurata*.

The method developed to process data was divided into two stages, as seen in the schematic diagram of Figure 3. The objective of the first stage was the localization of the interesting landmarks of fish eyes (pupil and iris) and the opercular spine. The objective of the second stage was to develop a freshness model by taking these different areas into account. Each stage is explained in detail in the following subsections:

#### 2.2.1. Stage 1: Determining Landmarks

The regions of interest for freshness evaluations in fish, as suggested by [11,12], are fish eyes and the zone that corresponds to the opercular spine after the ventral insertion of the pectoral fin. This spatial segmentation was performed manually by the expert operators of these zones, but these zones could be detected automatically from the 3D model. Specifically the fish eyes in this work were segmented automatically. Our method proved to be a more sophisticated way of differentiating between pupils and irises.

(1) Automatic Landmarking of Pupils and Irises

The automatic landmarking methods followed were based on spatial segmentation to find the position and radius of the pupils and irises of gilthead eyes. Spatial segmentation can be performed from different sources. Specifically in this case, spatial information was available from the hyperspectral camera, the RGB camera used for the structured light and the depth map built *D*. The spatial resolutions obtained from the RGB camera and the depth map were higher than from the hyperspectral camera. Thus three different segmentation methods were proposed based on the RGB image and depth map *D* for automatic pupil localizations. Having located pupils, the iris region was determined as the region that resulted from the difference between two circles: a circle with the same center and three times the size of the pupil circle and the circle pupil. This ratio was experimentally fixed because the distance between the camera and samples was constant.

The first segmentation method, based on the RGB image, was used to apply a circumference detector to the edges detected in the original image. Hough transform was used as a detection method and the average pixel intensity level of the internal circumference was employed to filter false-positives.

The second segmentation method based on the reconstructed depth map *D* easily detected gilthead pupils because they appear as a round hole on the reconstructed surface (Figure 3). This is because the laser light was unable to reflect when it fell on gilthead pupils as it penetrated eyes.

Finally, the third proposed method combined the two previous methods to enhance segmentation robustness. This approach was composed of an RGBD image by spatially relating both workspaces. Hence it was possible to apply the two previous methods to detect gilthead pupils.

To spatially relate the RGB image with depth map D, the RGB camera was calibrated in relation to the world reference system so that D could then be projected on the RGB camera to acquire the image that the camera would capture. Therefore, an RGBD image consisted of inputting the height of the reconstructed points as image data.

(2) Proposal for Automatic Landmarking of Opercular Spine Localization

**Figure 4:**
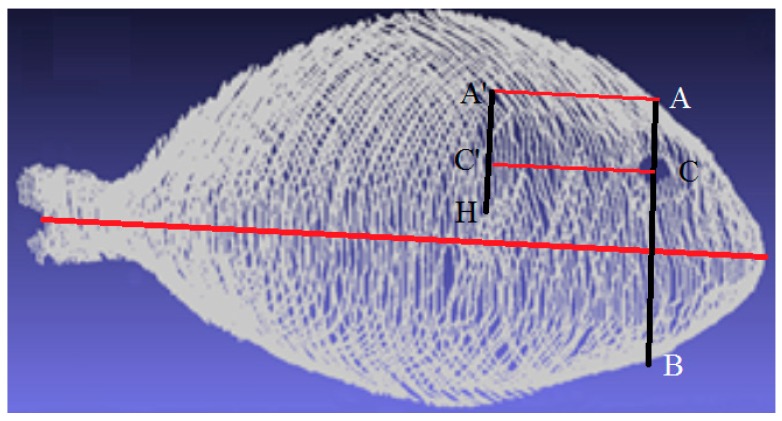
The 3D model image with the schematic method to landmark the opercular spine (A’C’) using the center of the eye (C) and highest point H.

The opercular spine region was marked manually in this study using the 3D model of the fish it would be easy to select also automatically in this region. For example, a proposed method for detecting the opercular spine could be (Figure 4):

1. The central axis is detected to take into account orientation changes.
2. The center of the eye is located by the previously explained techniques.
3. An imaginary profile AB, perpendicular to the central axis, is calculated that crosses the fish, as depicted in Figure 4.
4. The AC profile is found because AC is shorter than CB.
5. Highest point H is found.
6. The AC profile is projected in the direction of the central axis as a distance defined by H to obtain the A’C’ profile.
7. A region around the A’C’ profile is the opercular spine region.

(3) Manual Landmarking

A manual landmarking method was developed to gain a measure of the error of automatic landmarking and for opercular spine localization. This method was based on a graphical interface, which was developed in Matlab R2013a (The Mathworks, Natick, MA, USA), where operators could easily mark the lean after the opercular spine position and draw a circle (position and radius) on the fish eye using 2D color images. The obtained 2D image opercular spine positions (pixel coordinates) were transformed into the coordinate system of the hyperspectral system to acquire the spectral information of this region. The position and radius of fish eyes (also expressed in pixels) were converted into the International System of Units by the camera’s calibration model. It is stressed that the information of the position and radius of fish eyes was manually obtained and used only as ground truth to compare the automatic method results.

#### 2.2.2. Stage 2: Freshness Model

Freshness models were developed using multivariate analyses, specifically a partial least squares (PLS) regression model for each region defined in stage 1. The PLS technique models the relationship and structure between spectra and their time lapse. PLS is considered a basic tool in chenometrics [37], and is now widely employed. Specifically, the PLS implementation of the employed algorithm was SIMPLS [38], which calculates the PLS factors directly as linear combinations of the original variables. This algorithm was chosen because it is faster than NIPALS. The best region selection for freshness evaluations was based on the results of the developed PLS models. Smaller models were also developed by selecting the most influential wavelengths using the I-PLS forward algorithm of interval selection [39]. This algorithm selects a subset of variables for building the PLS model, which will give a similar prediction compared to using all the variables. I-PLS makes an exhaustive search for the best combination of variables.

### 2.3. Statistical Validation

Spatial segmentation was validated by calculating the error between the position (Equation (7)) and the radius (Equation (8)) of the automatic method with the measures obtained manually with the image tool.

(7) $$\mathrm{PE}=\overset{n}{\underset{i=1}{\sum }}\|\hat{p_{i}}-p_{i}\|$$

where PE is the position error, $\hat{p}$*_i_* are the center positions of the circles estimated by the automatic method (x,y,z), *p_i_* are the center positions of the manually marked circle, and *n* is the total number of samples in the data set.

(8) $$\mathrm{RE}=\sqrt{\frac{\sum_{i=1}^{n}{\left({\hat{r}}_{i}-r_{i}\right)}^{2}}{n}}$$

where RE is the radius error, $\hat{r}$*_i_* are the radii of the circles estimated by the automatic method, *r**_i_*** are the radii of the manually marked circle, and *n* is the total number of samples in the data set.

The average deviation of the freshness models, from the calibration data, was calculated by Root-Mean-Square Error of Calibration (RMSEC), defined as:

(9) $$\mathrm{RMSEC}=\sqrt{\frac{\sum_{i=1}^{n}{\left({\hat{y}}_{i}-y_{i}\right)}^{2}}{n}}$$

where $\hat{y}$i are the predicted variable values, *y_i_* are the known values, and *n* is the total number of objects in the data set. RMSECV and RMSEPred are calculated with the same equation but with the predicted variable values obtained from the cross-validation samples or from the prediction samples respectively.

Validation of the freshness models was performed by a cross-validation method (CV) and with external validation (Pred); 75% of the samples (36 samples) were used to do the method and 20% of them were employed in the CV. The CV method employed was “Random blocks” and was repeated three times. The cross-validation method was employed as suggested by [40] given the number of samples. The remaining 25% were used in the external validation (prediction).

All the statistical procedures were performed by PLS Toolbox 6.2 (Eigenvector Research Inc., Wenatchee, WA, USA), a toolbox extension that runs in the Matlab computational environment.

## 3. Results and Discussion

### 3.1. Chemical and Microbiological Results

Table 1 shows the chemical and microbiological results of the samples stored at 4 °C. No difference was reported for the TVB-N and pH values on the first three days, which agrees with results obtained by other authors [4,5,10].

The TVB-N content in the freshly caught fish typically fells between 5 and 20 mg/100 g, whereas levels of 30–35 mg·N/100 g were generally considered an acceptable limit for some fish species stored in ice (EEC, 1995).

Once again, no difference was observed for the RI values taken on Days 0, 1 and 3. However, the values increased with storage time, which agreed with the RI values established by [30] (lower than 1.3355 was very good, 1.3356–1.3365 good, 1.3366–1.3390 moderate, and over 1.3390 indicated spoilage).

Our microbiological counts agreed with other authors [10,41]. The values obtained on Day 6 for Enterobacteriaceae and mesophilic counts were lower than the limits allowed for human consumption, which are 7 log cfu/g and 6–7 log cfu·g/L, respectively [42,43,44,45], but agreed with the TVB-N and RI values.

### 3.2. Image Analysis

#### 3.2.1. Stage 1: Landmarks Determination

**Figure 5:**
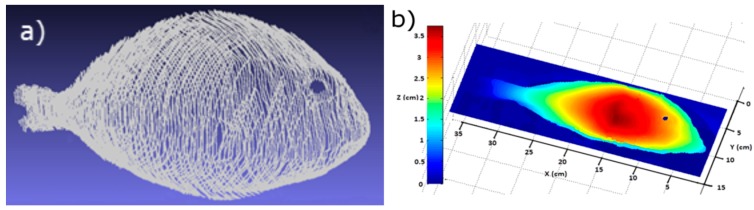
3D reconstruction of a gilthead using the 3D system based on structured light. (**a**) The 3D lines obtained directly from the structured light; (**b**) The 3D shaded surface built where color is proportional to surface height.

As the example in Figure 5 depicts, a high-resolution 3D whole gilthead reconstruction was achieved using the developed 3D SL acquisition system. Due to the fish’s hill shape, it was necessary to use two lasers to reconstruct the whole sample accurately. The resolutions reached were 3 × 10^−5^ m, 1 × 10^−4^ m and 103 × 10^−6^ m on the *X*-, *Y*- and *Z*-axis, respectively. Figure 5a depicts the reconstructed 3D points obtained by the laser, and a smoothed surface obtained from the 3D points is shown in Figure 5b. Any fish region can be automatically selected by using this 3D model.

**Figure 6:**
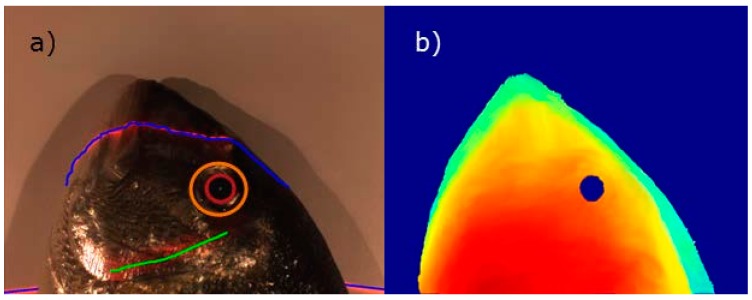
(**a**) RGB image of a gilthead. The red circumference marks the pupil detected by the Hough method and the orange circle marks the iris; (**b**) Composed image of the height projected using the RGB camera model with depth information. The gilthead pupil appears as a hole.

For the eye localization results, Figure 6a provides a qualitative example of the method used to detect circles by Hough transform in order to segment gilthead pupils. Thanks to the method developed to filter false-positives, only one circle appeared, which was well placed in the pupil (red circle). Then a bigger circle was created using the pupil as a base that matched the iris (orange circle). In Figure 6b, the method used to detect the pupil was based on 3D information, where the pupil was a round hole. Depth information was projected onto the RGB camera model. Thus the results of both methods were expressed in the same coordinate system and were used together to achieve more robust spatial segmentation. This combination could avoid, for example, false-positives from the 2D method with round dark spots on fish, and could also reduce noise in 3D information.

The eyes measured manually by the developed software had a pupil radius of 4 × 10^−3^ m ± 5 × 10^−4^ m (mean value plus/minus standard deviation), while radius of the whole eyes was 12 × 10^−3^ m ± 5 × 10^−4^ m. The proposed automatic method had a radius error (RE) of 4 × 10^−4^ m for pupil size, which represents about a 3.3% error. The position error of the automatic method was 2 × 10^−4^ m (PE), which only doubled the Y resolution of the 3D system. The iris had a similar error because it was calculated using the pupil position and the radius in a fixed manner. The grosser error in the radius was probably due to the error in the ground truth, specifically because people tended to make bigger circles than the correct size to ensure that pupils and eyes remained inside their circles, and that the centers of these circles were right. These errors were less serious than those obtained by [45] for automatic landmarking in sea bass.

#### 3.2.2. Stage 2: Freshness Evaluation

Table 2 shows the results of the PLS models used to predict freshness based on these regions. The opercular spine region had a much lower R^2^ than that reported by Menesatti (0.41 vs. 0.82) for sea bass. This finding indicated that this region was not useful for gilthead freshness estimations. These results are supported by those obtained by other authors [11], which affirmed that the regions of gills and eyes were the best indicated regions for gilthead freshness estimations. Using neural networks, Dowlati et al. obtained a lower RMSEPred in eyes (0.651 vs. 1.34) and a better R^2^ Pred (0.99 vs. 0.92) because they did the study over an 18-day period and the present was done on the six first days. The same occurred with the prediction of freshness with a potentiometric sensor made by [8], who obtained a R^2^ Pred of 0.96 for a 15-day period. The exponential degradation of fish means that it could be easier to predict its freshness over longer periods. In fact no significance signal was received by the potentiometric sensor [8] until storage Day 3. Furthermore, the best results in this study were obtained using the spectra of gilthead eyes, especially when employing only the pupil region. These results could be explained because pupils and irises had different spectra, which better fitted the PLS models when employed separately. The values of the results obtained when the pupil region was used were higher because pupils had a narrower variability than the irises between fish. Fish eyes tended to be white and cloudy during the storage period [11], especially pupils. This cloudy process meant major changes in the intensity of the reflected spectra, which correlated directly with freshness to explain why the PLS that used pupils achieved better results. Organoleptic changes, such as color and appearance in fish eyes after death, could be attributed to internal chemical variations [46]. The RMSEPred and RMSECV of the PLS model indicated that freshness can be predicted with an error of around one day for fish stored at 4 °C.

**Figure 7:**
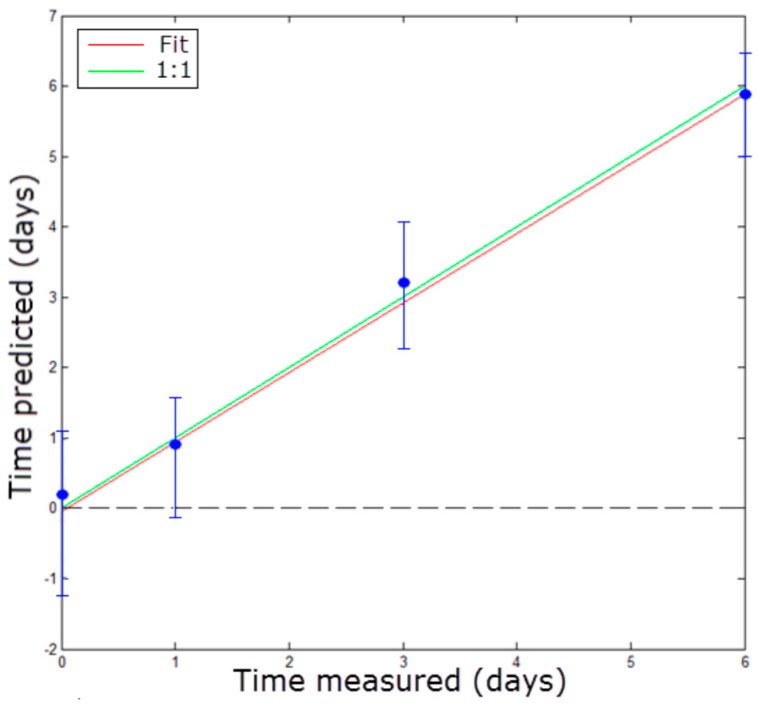
The PLS freshness prediction using the pupil spectra. Error bars correspond to the standard deviation of the predicted values in days.

The values predicted for the samples used to test the PLS model, and built using the pupil spectra, are plotted in Figure 7. This figure agrees with the RMSEPred values obtained (Table 2) and indicates that this model can predict freshness with an error of around one day for fish stored at 4 °C. It is important to note that not even the destructive analysis was able to differentiate among Days 0, 1 and 3.

A forward I-PLS model was calculated to obtain the best correlated wavelength. The I-PLS algorithm added wavelengths until the RMSECV improved no further, which were the following five wavelengths, 400, 411, 478, 680 and 982 nm, for the pupil spectra, and 400, 411, 489.6, 512 and 971 nm for the iris spectra. The importance of visible wavelengths was accounted for by the previously explained cloudy effect noted in fish eyes with time. The 971 nm wavelength corresponded to the absorption band of water, while the 982 nn band corresponded to the 2nd overtone of the O–H stretch. The changes in band 982 could be explained by the O–H stretch in hypoxanthine (C5H4N4O). Some authors [47] have shown that hypoxanthine linearly exhibits a postmortem increase in the vitreous humor concentration. We highlight that these five I-PLS selected wavelengths fell within the visible and near infrared range, which justified the spectral range employed herein. Employing only these wavelengths with pupils gave worse results (R^2^ Pred), only around 5.4% ((0.92–0.87) × 100/0.92), and reduced the model from 54 to 5 wavelengths. This reduced model could be interesting for some industrial applications in which processing time is essential.

## 4. Conclusions

This study focused on combining a 3D structured light system with a hyperspectral imaging system to solve the problem of non destructively estimating *Sparus aurata* (gilthead) freshness. This novelty approach allowed the hyperspectral inspection of very specific fish areas, where previous studies have shown that more information for freshness estimations can be acquired. The structured light-based 3D system conferred a high-resolution point cloud of fish, where the developed method automatically located gilthead pupils and irises with an error of 4 × 10^−4^ m and a radius of 2 × 10^−4^ m in the position. Only these regions were acquired by hyperspectral imaging within the short-wave near-infrared range, which demonstrated that enough information was provided for freshness estimations. This study confirmed the need for accurate robust spatial segmentation for freshness estimations by SW-NIR. The best region found was pupils with an R^2^ of 0.92 in the prediction done with a PLS model and using the entire wavelength range, and also one of 0.87 when employing only the five best correlated wavelengths. By way of conclusion, according to on the good results achieved, the combination of 3D technology and a hyperspectral analysis offers plenty of potential and can be considered a very promising technique to non destructively predict gilthead freshness with an error of only one day for the fish stored at 4 °C. Furthermore studies should be performed, employing different storage temperatures and biological analysis, in order to obtain more robust prediction models.

## Figures and Tables

**Table 1:** Chemical and microbiological results.

Storage Time (Days)	RI	TVB-N	pH	Enterobacteriaceae (log cfu)	Mesophilic (log cfu)
0	1.3348 ± 0.0003 a	18.14 ± 2.25 a	6.28 ± 0.18 a	<1	0.37 ± 0.74
1	1.3350 ± 0.0002 a	18.57 ± 0.61 a	6.11 ± 0.04 ab		
3	1.3352 ± 0.0006 a	19.47 ± 1.79 a	6.22 ± 0.108 ab		
6	1.3371 ± 0.0010 b	28.06 ± 1.29 b	6.23 ± 0.09 b	3.30 ± 0.22	5.02 ± 0.24

Measured values are the average of three analyses ± standard deviation. a–b, The difference with the different superscript letter in the same column is significant at the 0.05 level (2-tailed).

**Table 2:** The PLS results for predicting gilthead freshness.

	Iris		Pupil		Pupil & Iris	Opercular Spine
	Whole Spectrum	I-PLS Selection	Whole Spectrum	I-PLS Selection	Whole Spectrum	Whole Spectrum
Num. LVs	7	3	7	3	5	6
RMSEC (days)	0.805	1.041	0.604	0.908	1.174	0.662
RMSECV (days)	0.968	1.071	0.712	0.941	1.212	0.803
RMSEPred (days)	0.882	0.971	0.651	0.846	1.253	0.783
R^2^ Cal	0.87	0.79	0.93	0.84	0.74	0.566
R^2^ CV	0.82	0.77	0.90	0.82	0.72	0.391
R^2^ Pred	0.86	0.83	0.92	0.87	0.7	0.413
